# Evaluating the Accuracy of Artificial Intelligence (AI)-Generated Illustrations for Laser-Assisted In Situ Keratomileusis (LASIK), Photorefractive Keratectomy (PRK), and Small Incision Lenticule Extraction (SMILE)

**DOI:** 10.7759/cureus.67747

**Published:** 2024-08-25

**Authors:** Dallas J Petroff, Ayesha A Nasir, Kayvon A Moin, Bosten A Loveless, Omeed A Moshirfar, Phillip C Hoopes, Majid Moshirfar

**Affiliations:** 1 Ophthalmology, Idaho College of Osteopathic Medicine, Meridian, USA; 2 Ophthalmology, University of Louisville School of Medicine, Louisville, USA; 3 Ophthalmology, Hoopes Vision Research Center, Hoopes Vision, Draper, USA; 4 Medicine, American University of the Caribbean, Cupecoy, SXM; 5 Ophthalmology, Rocky Vista University College of Osteopathic Medicine, Ivins, USA; 6 Sam Fox School of Design and Visual Art, Washington University in St. Louis, St. Louis, USA; 7 Ophthalmology, John A. Moran Eye Center, University of Utah School of Medicine, Salt Lake City, USA; 8 Eye Banking and Corneal Transplantation, Utah Lions Eye Bank, Murray, USA

**Keywords:** klex, astigmatism, generative ai model, eye, cornea, ophthalmology, myopia, corneal refractive surgery, medical illustration, artificial intelligence

## Abstract

Purpose: To utilize artificial intelligence (AI) platforms to generate medical illustrations for refractive surgeries, aiding patients in visualizing and comprehending procedures like laser-assisted in situ keratomileusis (LASIK), photorefractive keratectomy (PRK), and small incision lenticule extraction (SMILE). This study displays the current performance of two OpenAI programs in terms of their accuracy in common corneal refractive procedures.

Methods: We selected AI image generators based on their popularity, choosing Decoder-Only Autoregressive Language and Image Synthesis 3 (DALL-E 3) for its leading position and Medical Illustration Master (MiM) for its high engagement. We developed six non-AI-generated prompts targeting specific outcomes related to LASIK, PRK, and SMILE procedures to assess medical accuracy. We generated images using these prompts (18 total images per AI platform) and used the final images produced after the sixth prompt for this study (three final images per AI platform). Human-created procedural images were also gathered for comparison. Four experts independently graded the images, and their scores were averaged. Each image was evaluated with our grading system on “Legibility,” “Detail & Clarity,” “Anatomical Realism & Accuracy,” “Procedural Step Accuracy,” and “Lack of Fictitious Anatomy,” with scores ranging from 0 to 3 per category allowing 15 points total. A score of 15 points signifies excellent performance, indicating a highly accurate medical illustration. Conversely, a low score suggests a poor-quality illustration. Additionally, we submitted the same AI-generated images back into Chat Generative Pre-Trained Transformer-4o (ChatGPT-4o) along with our grading system. This allowed ChatGPT-4o to use and evaluate both AI-generated and human-created images (HCIs).

Results: In individual category scoring, HCIs significantly outperformed AI images in legibility, anatomical realism, procedural step accuracy, and lack of fictitious anatomy. There were no significant differences between DALL-E 3 and MiM in these categories (p>0.05). In procedure-specific comparisons, HCIs consistently scored higher than AI-generated images for LASIK, PRK, and SMILE. For LASIK, HCIs scored 14 ± 0.82 (93.3%), while DALL-E 3 scored 4.5 ± 0.58 (30%) and MiM scored 4.5 ± 1.91 (30%) (p<0.001). For PRK, HCIs scored 14.5 ± 0.58 (96.7%), compared to DALL-E 3's 5.25 ± 1.26 (35%) and MiM's 7 ± 3.56 (46.7%) (p<0.001). For SMILE, HCIs scored 14.5 ± 0.68 (96.7%), while DALL-E 3 scored 5 ± 0.82 (33.3%) and MiM scored 6 ± 2.71 (40%) (p<0.001). HCIs significantly outperformed AI-generated images from DALL-E 3 and MiM in overall accuracy for medical illustrations, achieving scores of 14.33 ± 0.23 (95.6%), 4.93 ± 0.69 (32.8%), and 5.83 ± 0.23 (38.9%) respectively (p<0.001). ChatGPT-4o evaluations were consistent with human evaluations for HCIs (3 ± 0, 2.87 ± 0.23; p=0.121) but rated AI images higher than human evaluators (2 ± 0 vs 1.07 ± 0.73; p<0.001).

Conclusion: This study highlights the inaccuracy of AI-generated images in illustrating corneal refractive procedures such as LASIK, PRK, and SMILE. Although the OpenAI platform can create images recognizable as eyes, they lack educational value. AI excels in quickly generating creative, vibrant images, but accurate medical illustration remains a significant challenge. While AI performs well with text-based actions, its capability to produce precise medical images needs substantial improvement.

## Introduction

With the prevalence of myopia approaching 50% globally, corneal refractive procedures are gaining popularity [1]. Some options are laser-assisted in situ keratomileusis (LASIK), photorefractive keratectomy (PRK), and small incision lenticule extraction (SMILE). These procedures are commonly available to correct myopia, hyperopia, and astigmatism. In LASIK, a corneal flap is created with a femtosecond laser, followed by ablation of corneal tissue with an excimer laser [2]. During PRK, the corneal epithelium is debrided, the underlying stroma is ablated with an excimer laser, and a bandage contact lens is placed for appropriate wound healing [3]. In SMILE, a femtosecond laser is used to create a corneal lenticule that is extracted for refractive error correction [4].

Given the current popularity of artificial intelligence (AI), we were interested in ways AI can be utilized to help patients understand their medical procedures. Other specialties like dermatology and radiology are testing AI's potential in image recognition, discovering that further improvements are needed [5-8]. Other sources show that while AI can create images, the accuracy of said images is questionable [9-11]. Notable AI programs like decoder-only autoregressive language and image synthesis 3 (DALL-E 3) [12] and chat generative pre-trained transformer-4o (ChatGPT-4o) [13] are at the forefront of AI image-creating advancements. Other programs like Midjourney for image creation are still in early development and are not yet available for free public use [14]. These programs allow users to address niche problems or generate images from simple prompts, offering limitless possibilities, such as creating diagrams to help patients understand their upcoming procedures.

In this study, we use DALL-E 3 and medical illustration master (MiM) [15] to create images describing LASIK, PRK, and SMILE. Our goal is to assess and determine the reliability, credibility, and accuracy of the generated images for educational use via a created objective grading system.

## Materials and methods

Study design

This study adhered to the tenets of the Declaration of Helsinki and the Hoopes Vision Ethics Committee and the Biomedical Research Alliance of New York (BRANY) Institutional Review Board (IRB) approved this study procedure (#A20-12-547-823).

We selected AI image generators based on their current popularity. DALL-E 3 was chosen for its leading position in image generation, and MiM was discovered through OpenAI’s search function. Using the phrase “Medical Illustrations,” we filtered 11 responses by comment and download count, selecting MiM for its high engagement [15].

**Figure 1:**
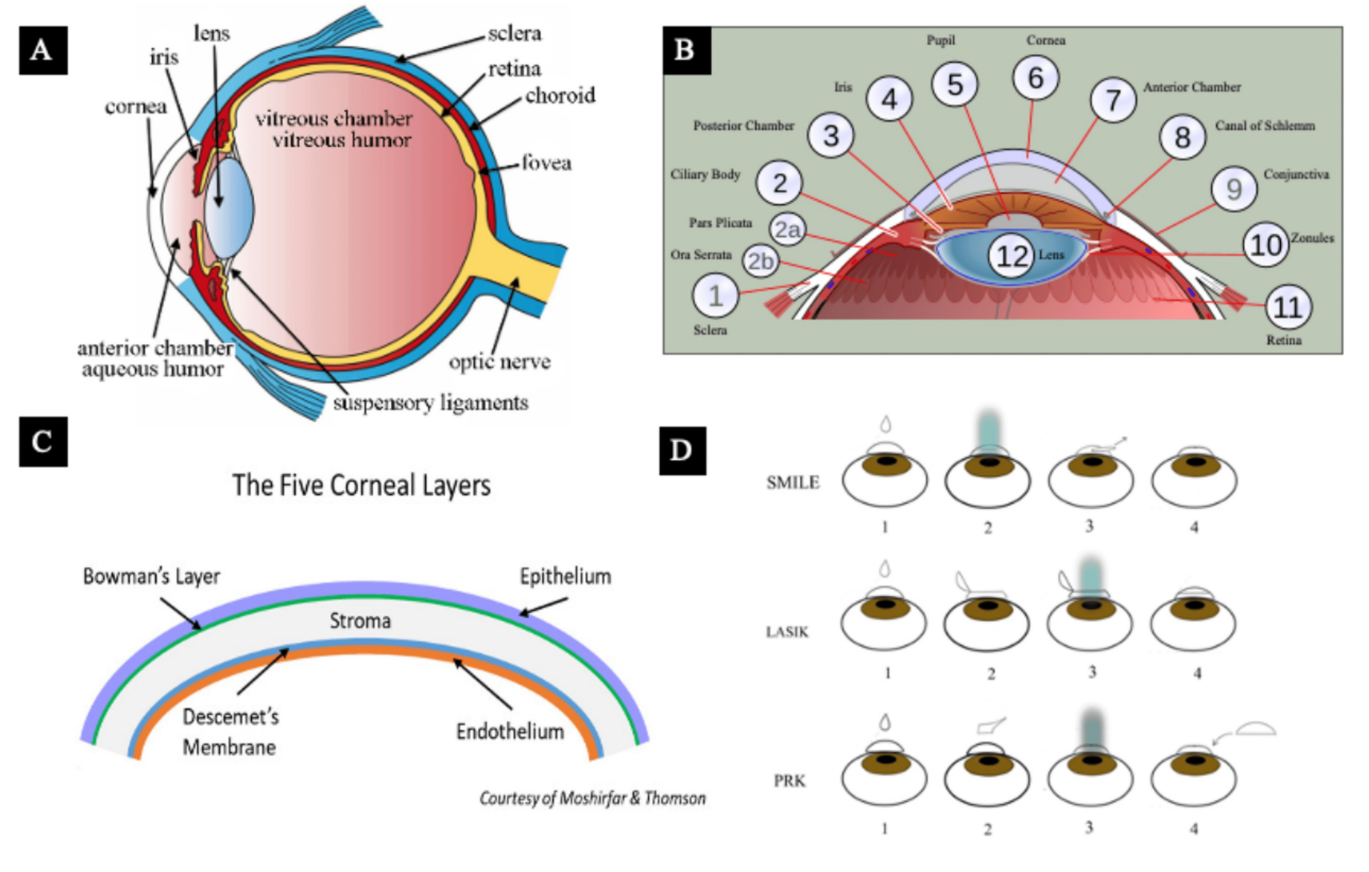
Administered AI prompts with the corresponding explanation (A) Ocular anatomy, (B) anatomy of the anterior segment, (C) cross-section of the cornea, and (D) basic procedural diagram of refractive procedures in question. The goal of this figure was to judge the AI’s ability to interpret an externally uploaded image and modify its generated illustration to match a given template. Copyright/license: (A, B) have been adapted from an open-access source, courtesy of Dr. Holly Fischer [16] and Dr. Jordi Nogue [17], respectively. (C) has been adapted from an open-source book [18] distributed under the terms of the Creative Commons Attribution-NonCommercial-NoDerivatives 4.0 International (CC BY-NC-ND 4.0) (http://creativecommons.org/licenses/by-nc-nd/4.0/). (D) was created by the authors with Adobe Illustrator. Abbreviations: SMILE = small incision lenticule extraction; LASIK = laser-assisted in situ keratomileusis; PRK = photorefractive keratectomy

**Figure 2:**
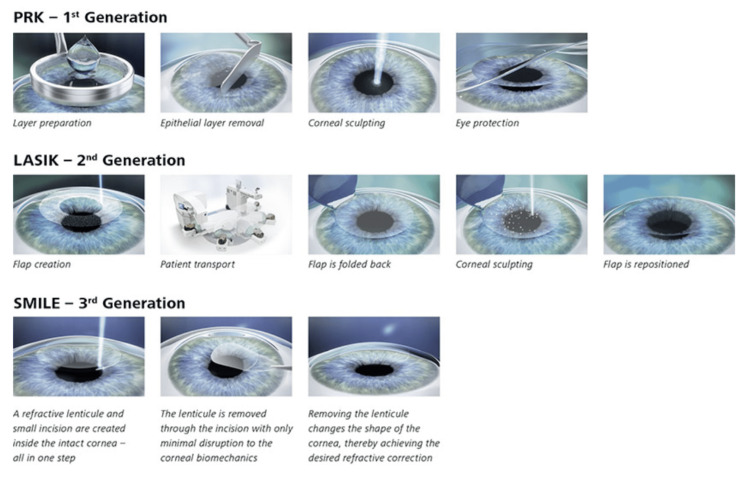
Human-created images (HCIs) for PRK, LASIK, and SMILE The following figure depicts a simplified schematic of the three tested corneal refractive procedures LASIK, PRK, and SMILE with brief captions expanding upon each step. Permission for use of this image was obtained from the publishers [19]. Abbreviations: PRK = photorefractive keratectomy; LASIK = laser-assisted in situ keratomileusis; SMILE = small incision lenticule extraction

In this study, we employed six self-created prompts to generate images using DALL-E 3 and MiM to depict the refractive procedures LASIK, PRK, and SMILE (Table 1). These prompts were specifically designed to evaluate various aspects of AI image creation and modification. The prompts were used sequentially, with a new chat session created between procedures to avoid memory retention bias. Throughout this study, we produced over 36 images. However, we only considered the final images generated after the sixth prompt, resulting in six final images for evaluation. The image used to examine DALL-E 3 and MiM’s ability to format to an example can be seen in Figures 1A-1D [16-18]. We conducted an internet search to locate human images that accurately represented the procedures employed in our study. These Human-Created Images (HCIs) were used as a comparison to evaluate against the AI-generated images of the same procedures (Figure 2) [19].

**Table 1:** Administered AI prompts with the corresponding explanation Abbreviations: AI = artificial intelligence

Administered AI prompts	Purpose of prompt
1. Create a medical illustration for the corneal refractive procedure in question while ensuring the medical illustration is anatomically and surgically accurate.	1. Determine if AI is able to discern a medically accurate response and acknowledgement of prompt.
2. Depict all surgical steps in a sequential fashion, with each step portrayed onto one singular eye.	2. Evaluate programs’ understanding of each surgical procedural step and ability to display it accurately in sequential order.
3. For each surgical step, include a brief caption summarizing each step and place it within the image.	3. Assess the ability of an AI model to recognize specific individual steps from an illustration, develop an accurate explanation that describes each step, and to integrate captions seamlessly into the illustration.
4. Make each depicted iris purple while maintaining image clarity and legibility.	4. Test the AI programs’ ability to recognize a specific part of ocular anatomy from a fabricated illustration, then enact a modification on said part.
5. Adjust the formatting to match the following example (Figures 1A-1D)	5. Judge ability of AI programs to interpret an uploaded image and modify its fabricated illustration to match the provided image template.
6. Condense the image to include only the three most pertinent steps of the procedure while retaining captions describing the relevant steps.	6. Gauge comprehension skills of the AI program, examining its ability to condense information whilst maintaining utility.

**Figure 3:**
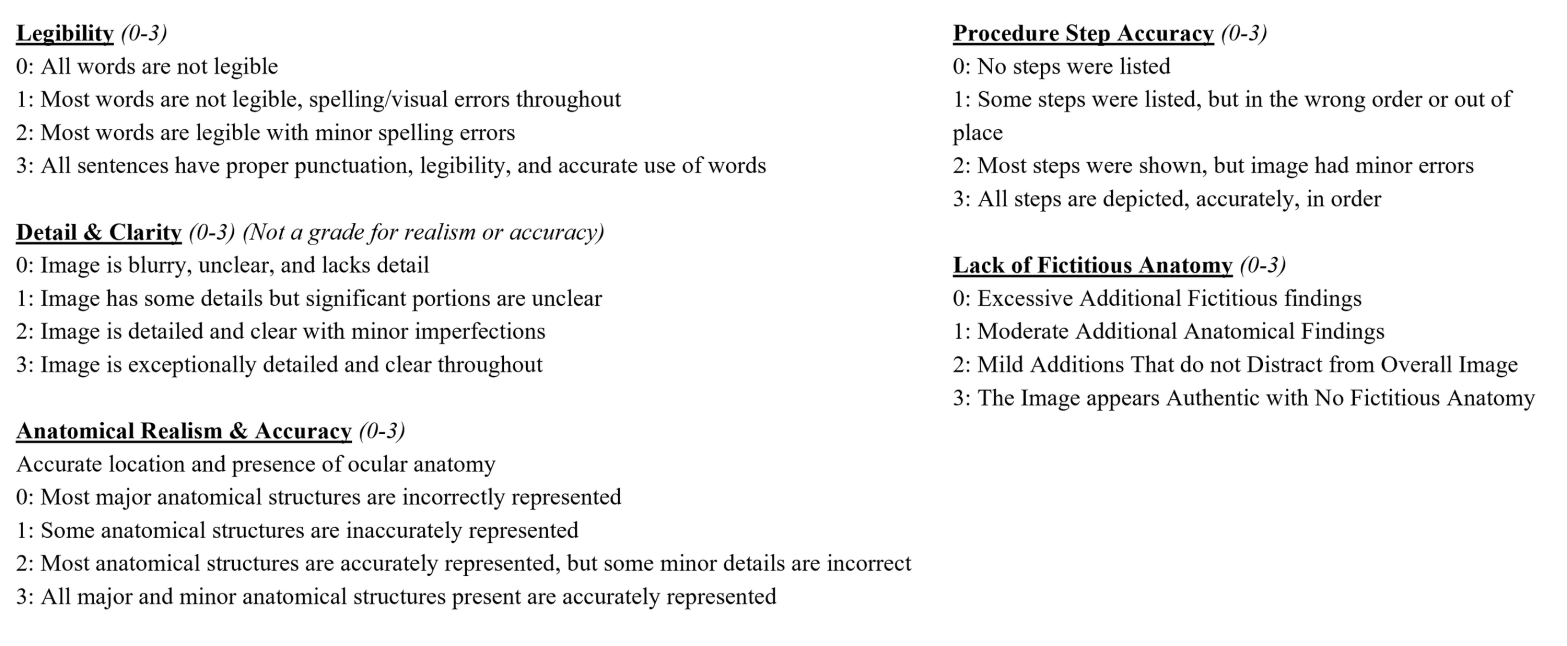
Objective grading system Used to grade each resultant AI-generated image and human-created image (HCI) Abbreviations: AI = artificial intelligence

All images were subsequently assessed by four corneal refractive surgeons at Hoopes Vision, each with over 10 years of experience using our developed grading system. The individuals were blinded to what platform they were assessing (DALL-E 3, MiM, or the HCIs). Our grading system was developed to objectively evaluate the final images, consisting of five categories: “Legibility,” “Detail & Clarity,” “Anatomical Realism & Accuracy,” “Procedural Step Accuracy,” and “Lack of Fictitious Anatomy.” Each category could earn up to three points, allowing for a maximum total of 15 points per image. A score of zero indicated an image with no educational value, while a score of 15 represented the performance of a highly accurate medical illustration that is both anatomically and procedurally correct. Figure 3 showed a further breakdown of scoring. This scoring system ensured a thorough evaluation of the images' educational utility.

Additionally, we used ChatGPT-4o to evaluate all the images using the same grading system applied by the human evaluators. To accomplish this, we initiated new chat sessions, uploaded each image along with the grading criteria, and asked ChatGPT-4o to evaluate the images. This allowed us to compare the differences in image assessment between AI and human evaluators.

Statistical analysis

Statistical analysis was conducted using Excel (Microsoft Corporation, Redmond, WA, USA). To compare the mean numerical scores of the control illustrations, DALL-E 3, and MiM for all three corneal refractive procedures, we employed a one-factor analysis of variance (ANOVA) with post hoc Tukey testing. Welch’s two-sample t-test for unequal standard errors was used to compare mean scores between reviewers and ChatGPT-4o for assessing AI’s grading performance. A threshold of 0.05 was used to define statistical significance for reported observations. Levene's test was applied to assess the equality of variances, and the population's variances were considered equal (p=0.09). The normality assumption was evaluated using the Shapiro-Wilk test (α = 0.05), and due to the small sample size, the distributions were deemed not normal. However, the ANOVA test is robust to moderate violations of the normality assumption. Post hoc power analysis for mean cumulative scores demonstrated a statistical power of 0.285.

## Results

**Figure 4:**
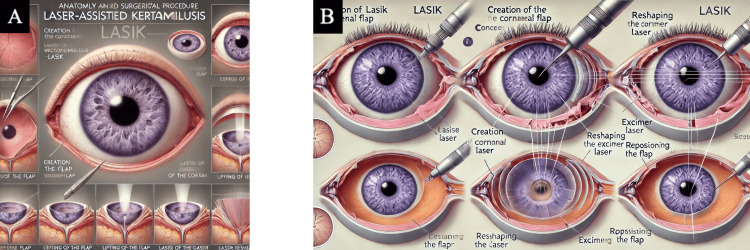
Laser-assisted in situ keratomileusis (LASIK) LASIK medical illustrations were generated by AI-powered text-to-image generators after inputting final prompt number six (Table 1). (A) Final image generated by DALL-E 3 [12]. (B) Final image generated by MiM [15]. Abbreviations: LASIK = laser-assisted in situ keratomileusis; DALL-E 3 = decoder-only autoregressive language and image synthesis 3; MiM = medical illustration master

**Figure 5:**
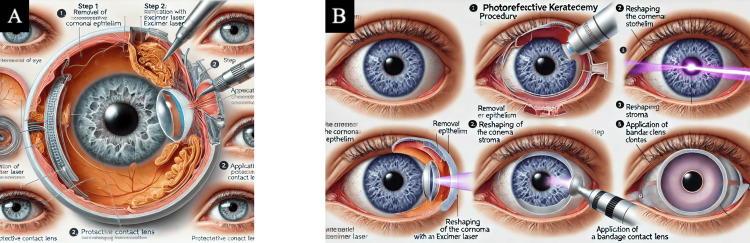
Photorefractive keratectomy (PRK) PRK medical illustrations were generated by AI-powered text-to-image generators after inputting final prompt number six (Table 1). (A) Final image generated by DALL-E 3 [12]. (B) Final image generated by MiM [15]. Abbreviations: PRK = photorefractive keratectomy; DALL-E 3 = decoder-only autoregressive language and image synthesis 3; MiM = medical illustration master

**Figure 6:**
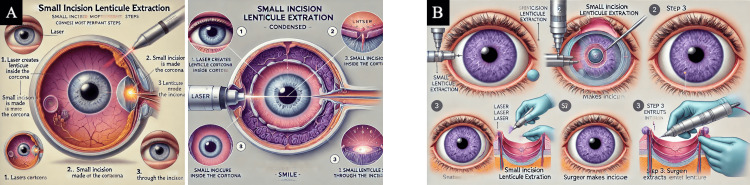
Small incision lenticule extraction (SMILE) SMILE medical illustrations were generated by AI-powered text-to-image generators after inputting final prompt number six (Table 1). (A) Final image generated by DALL-E 3 [12]. (B) Final image generated by MiM [15]. Abbreviations: SMILE = small incision lenticule extraction; DALL-E 3 = decoder-only autoregressive language and image synthesis 3; MiM = medical illustration master

Figures 4-6 showed the resultant AI images generated for each refractive procedure.

Observational findings

Out of the six final produced images, words were often illegible or misspelled, and the majority of the images included multiple instruments entering the eye inaccurately. For instance, Figure 4A showed a laser probe inside the anterior chamber, and images from each AI platform produced an iris and pupil within the vitreous cavity (Figures 4B, 5A, 6A). Neither platform could produce an illustration that displayed the surgeries in a procedural order. There was redundancy in step counts in images, and they were not accurate to the actual steps shown. Additionally, some figures created non-existent anatomy, such as a thickened choroid, the optic nerve originating from the lens, and extra organs stemming from the retina (Figures 5B, 6A, 6B).

Additionally, we provided the AI-generated images from this study to ChatGPT-4o for evaluation. It was able to accurately discern the content of each image and detail the procedures in order as they occurred in the image. However, when asked to create an image based on that same text, the resulting images were creative yet illogical.

Individual category scoring

**Figure 7:**
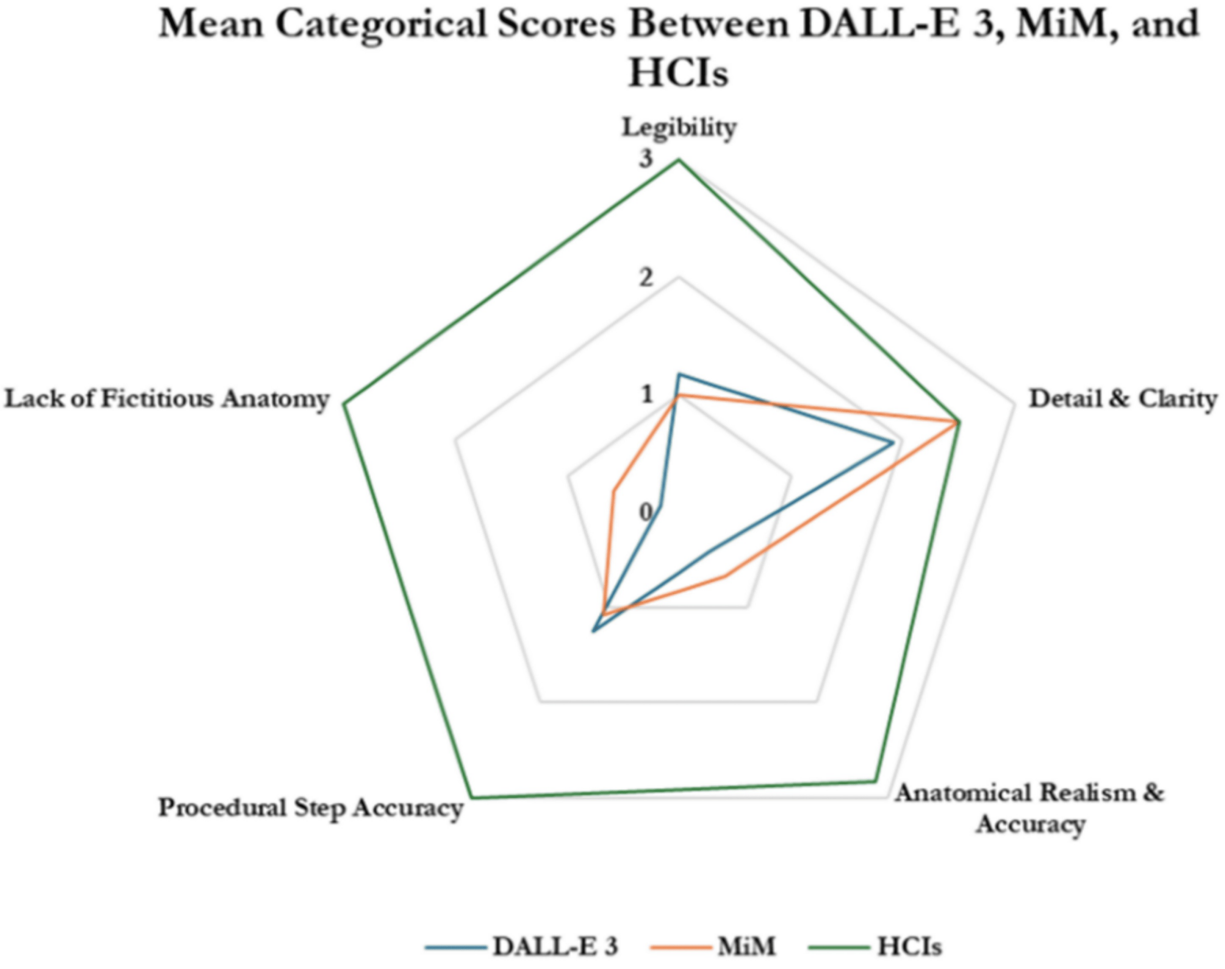
Radial diagram depicting mean categorical scores between DALL-E 3, MiM, and HCIs Abbreviations: DALL-E 3 = decoder-only autoregressive language and image synthesis 3; MiM = medical illustration manager; HCIs = human-created images

Starting with the legibility category, HCIs had significantly higher scores than images produced by DALL-E 3 and MiM (3.00 ± 0.00 vs. 1.17 ± 0.14 and 1.00 ± 0.25; p<0.001) (Table 2, Figure 7). There were no significant differences in legibility scores between DALL-E 3 and MiM (1.17 ± 0.14 vs. 1.00 ± 0.25; p=0.09). Images from all three groups had similar scores in detail & clarity, with the HCIs, DALL-E 3, and MiM receiving scores of 2.50 ± 0, 1.92 ± 0.52, and 2.50 ± 0.25, respectively; p>0.05). For anatomical realism & accuracy, the HCIs scored higher than DALL-E 3 and MiM (2.83 ± 0.29 vs. 0.42 ± 0.14 and 0.67 ± 0.29; p<0.001); There was no significant difference between DALL-E 3 and MiM (0.42 ± 0.14 vs. 0.67 ± 0.29; p=0.21). In the procedural step accuracy category, the HCIs scored higher than DALL-E 3 and MiM (3.00 ± 0 vs. 1.25 ± 0.25 and 1.08 ± 0.29; p<0.001). There were no significant differences in procedural step accuracy scores between DALL-E 3 and MiM (1.25 ± 0.25 vs. 1.08 ± 0.29; p=0.26). For the lack of fictitious anatomy category, HCIs scored higher than DALL-E 3 and MiM (3.00 ± 0.00 vs. 0.17 ± 0.14 and 0.58 ± 0.29; p<0.001). There were no significant differences in the lack of fictitious anatomy scores between DALL-E 3 and MiM (0.17 ± 0.14 vs. 0.58 ± 0.29; p=0.10).

**Table 2:** Mean categorical scores of DALL-E 3, MIM, and HCIS assigned by human reviewers Values are represented as mean ± standard deviation. Statistical significance was determined with Analysis of Covariance (ANOVA) with post hoc Tukey testing at p<0.05. * DALL-E 3 vs. MiM ** DALL-E 3 vs. HCI *** MiM vs. HCI Abbreviations: DALL-E 3 = decoder-only autoregressive language and image synthesis 3; MiM = medical illustration manager; HCI = human-created image

	DALL-E 3	MiM	HCIs	P*	P**	P***
Legibility	1.17 ± 0.14	1 ± 0.25	3 ± 0	0.09	0.001	0.003
Detail & clarity	1.92 ± 0.52	2.5 ± 0.25	2.5 ± 0	0.1	0.096	0.5
Anatomical realism & accuracy	0.42 ± 0.14	0.67 ± 0.29	2.83 ± 0.29	0.21	0.004	0.003
Procedural step accuracy	1.25 ± 0.25	1.08 ± 0.52	3 ± 0	0.26	0.003	0.012
Lack of fictitious anatomy	0.17 ± 0.14	0.58 ± 0.29	3 ± 0	0.1	< 0.001	0.002

Comparison between procedures

**Figure 8:**
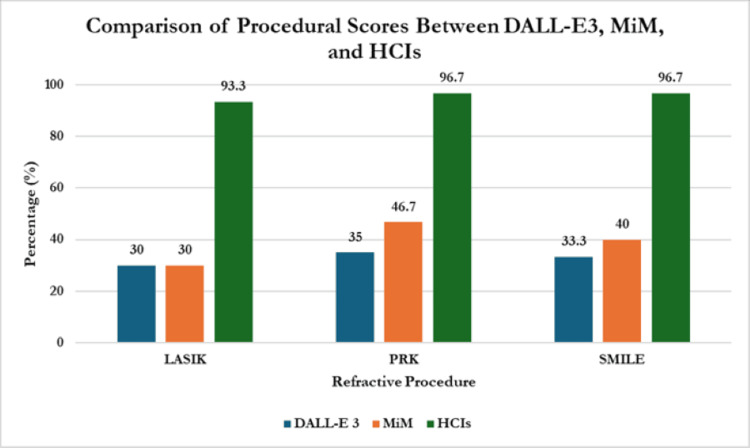
Comparison of overall score percentages between DALL-E 3, MiM, and HCIs Abbreviations: LASIK = laser-assisted in situ keratomileusis; PRK = photorefractive keratectomy; SMILE = small incision lenticule extraction; DALL-E 3 = decoder-only autoregressive language and image synthesis 3; MiM = medical illustration manager; HCIs = human-created images

When evaluating LASIK’s performance, the mean cumulative scores of DALL-E 3, MiM, and HCIs were 4.5 ± 0.58, 4.5 ± 1.91, and 14 ± 0.82, respectively. The mean scores out of 15 equated as a percentage are 30%, 30%, and 93.3%, respectively (Table 3, Figure 8). HCIs scored significantly better than DALL-E 3 and MiM (p<0.001); however, there was no statistical significance between DALL-E 3 and MiM mean scores (4.5 ± 0.58 vs. 4.5 ± 1.91; p=0.68).

**Table 3:** Mean cumulative scores of DALL-E 3, MiM, and HCIs assigned by human reviewers Values were represented as mean ± standard deviation. Statistical significance was determined with Analysis of Covariance (ANOVA) with post hoc Tukey testing at p<0.05. * HCI score significantly higher than DALL-E 3 and MiM scores Abbreviations: LASIK = laser-assisted in situ keratomileusis; PRK = photorefractive keratectomy; SMILE = small incision lenticule extraction; DALL-E 3 = decoder-only autoregressive language and image synthesis 3; MiM = medical illustration manager; HCIs = human-created images

	DALL-E 3	MiM	HCIs	P
LASIK	4.5 ± 0.58	4.5 ± 1.91	14 ± 0.82	<0.001*
PRK	5.25 ± 1.26	7 ± 3.56	14.5 ± 0.58	<0.001*
SMILE	5 ± 0.82	6 ± 2.71	14.5 ± 0.58	<0.001*
Mean cumulative score	4.93 ± 0.38	5.83 ± 1.26	14.33 ± 0.28	<0.001*

Evaluating PRK’s performance, the mean cumulative score of DALL-E 3, MiM, and HCIs were 5.25 ± 1.26, 7 ± 3.56, and 14.5 ± 0.58, respectively. The mean scores out of 15 equated as a percentage are 35%, 46.7%, and 96.7%, respectively. HCIs scored significantly better than DALL-E 3 and MiM (p<0.001); however, there was no statistical significance between DALL-E 3 and MiM mean scores (5.25 ± 1.26 vs. 7 ± 3.56; p=0.53).

Concerning SMILE’s performance, the mean cumulative scores of DALL-E 3, MiM, and HCIs were 5 ± 0.82, 6 ± 2.71, and 14.5 ± 0.68, respectively. The mean scores out of 15 equated as a percentage are 33.3%, 40%, and 96.7%, respectively. HCIs scored significantly better than DALL-E 3 and MiM (p<0.001); however, there was no statistical significance between DALL-E 3 and MiM mean scores (5 ± 0.82 vs. 6 ± 2.71; p=1.0).

When evaluating overall mean scores by procedure, PRK consistently outperformed SMILE and LASIK across all groups, but this was statistically insignificant (8.92 ± 4.64 vs. 8.5 ± 4.70 and 7.67 ± 4.81 respectively; p=0.81). Their mean scores as a percentage are 51% for LASIK, 59% for PRK, and 57% for SMILE over each AI platform.

Overall performance and scoring

**Figure 9:**
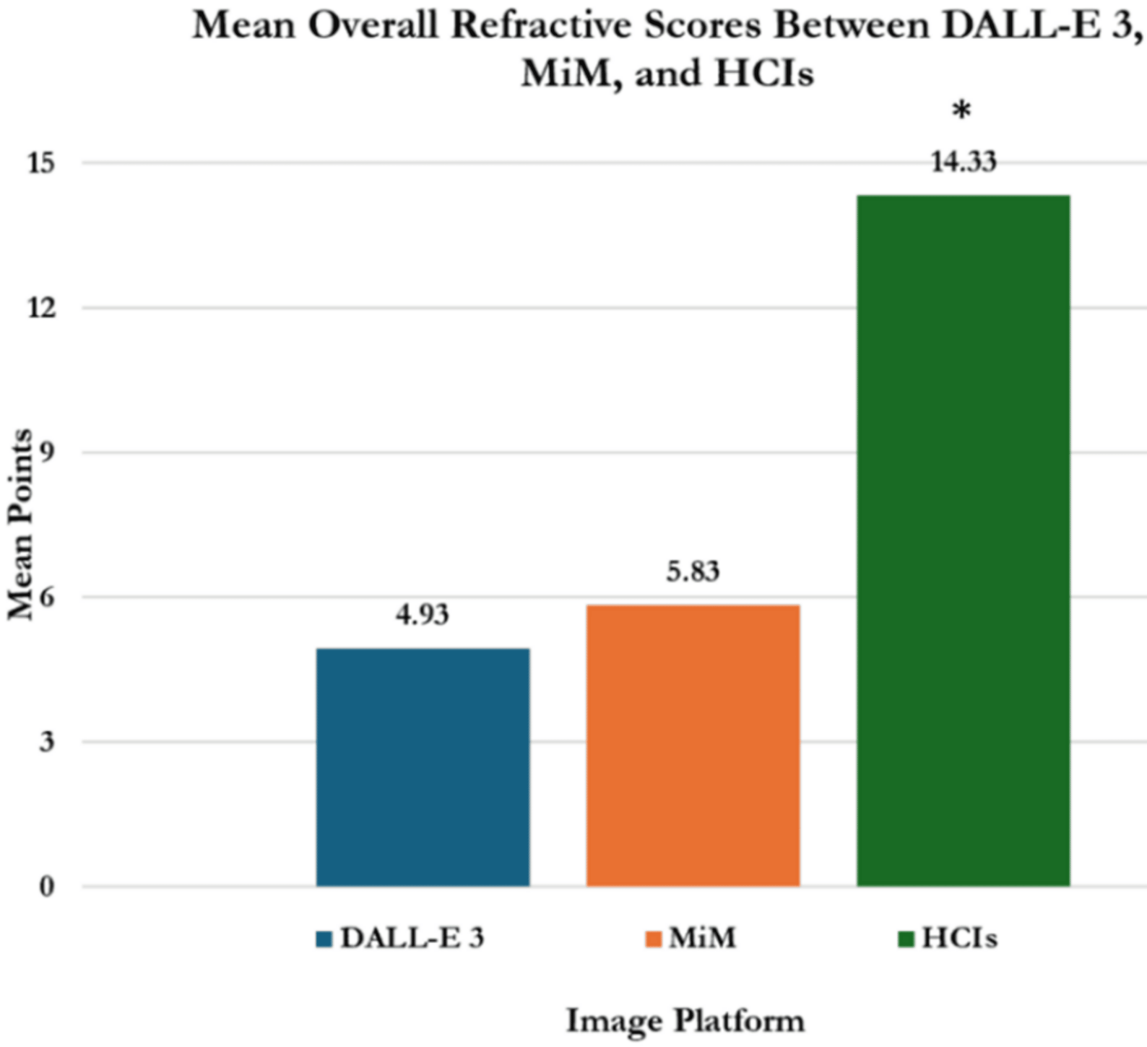
Overall mean scores earned per image using DALL-E 3, MiM, and HCIs Statistical significance was determined with Analysis of Covariance (ANOVA) with post hoc Tukey testing at p<0.05. * HCI score significantly higher than DALL-E 3 and MiM scores (p<0.001) Abbreviations: DALL-E 3 = decoder-only autoregressive language and image synthesis 3; MiM = medical illustration manager; HCIs = human-created images (HCI)

Using overall scores, HCIs significantly outperformed those generated by DALL-E 3 and MiM (14.33 ± 0.23 vs. 4.93 ± 0.69 and 5.83 ± 0.23, respectively; p<0.001) (Table 3, Figure 9). The mean scores equated to an overall score of 95.6% for human images, 32.8% for DALL-E 3, and 38.9% for MiM using 15 points as the potential total. MiM outperformed DALL-E 3 in mean score, but the difference was not significant (1.17 ± 0.77 vs 0.98 ± 0.69; p=0.7). Human images statistically outperformed DALL-E 3 images with mean scores of 2.87 ± 0.23 vs. 0.98 ± 0.69 (p<0.001). Human images also outperformed MiM images with mean scores of 2.87 ± 0.23 vs. 1.17 ± 0.77; p<0.001).

**Figure 10:**
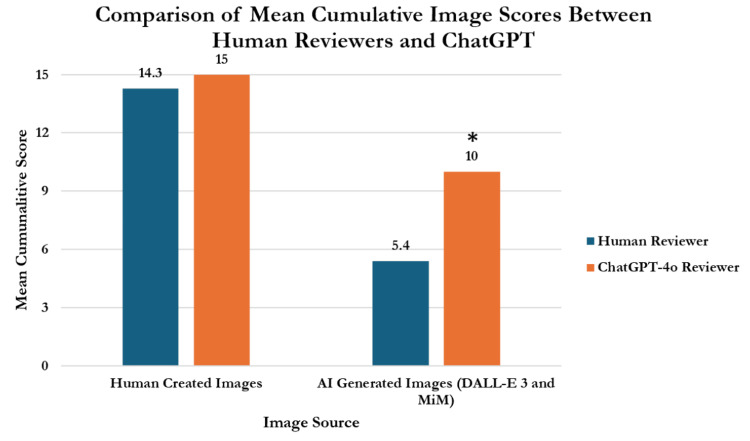
Comparison of grading performance between human reviewers and ChatGPT Statistical significance was determined with Welch's two sample t-test at p<0.05. *ChatGPT score significantly higher than human reviewer score (p<0.001) Abbreviations: DALL-E 3: decoder-only autoregressive language and image synthesis 3; MiM = medical illustration manager

When using ChatGPT-4o to evaluate images using our grading system, there was no significant difference between human and ChatGPT-4o evaluations of HCIs (2.87 ± 0.23 vs. 3 ± 0; p=0.121) (Figure 10). However, ChatGPT-4o graded the AI images created by DALL-E 3 and MiM significantly better than the human evaluators (2 ± 0 vs 1.07 ± 0.73; p<0.001).

## Discussion

AI is being utilized to help advance image recognition in the medical field, providing benefits for diagnosis and treatment. It is enhancing the accuracy and efficiency of medical imaging tasks like providing early COVID-19 diagnosis off of CT images or recognizing diabetic retinopathy based on images alone [20,21]. Another study explored AI's potential in generating medical illustrations with a focus on Horner syndrome and hypothyroidism [22]. However, AI's potential for creating medically accurate illustrations, particularly in the field of ophthalmology, has been scarcely explored. We designed this study to evaluate the effectiveness of AI in creating images for refractive procedures, aiming to enhance education for both patients and healthcare providers.

Our study revealed that ChatGPT-4 significantly inflated its scores when grading AI-generated images using our custom grading system compared to independent reviewers. However, there was no significant difference between the scores given by ChatGPT-4 and the reviewers for control images created by the authors. The current literature lacks studies on AI's assessment of its own generated imagery. A study by Azad et al. found that human graders rated students' computer science questions as 51% correct, while AI graders rated them 89% correct, with a 12% false positive rate [23]. A plausible explanation for ChatGPT-4's inflated scores in our study is its inherent bias in favor of AI-generated images, contrasted by potential human bias against them. Additionally, AI appears capable of interpreting images based on minimal clues, which might confuse even those experienced in the field. Our study found that the AI platforms can produce detailed text descriptions, but fail in visual accuracy, highlighting current limitations in AI-generated medical illustrations. Each prompt input into the AI programs generated text responses that accurately portrayed the procedures mentioned. However, the resulting images did not reflect the text illustrating the procedure. For instance, DALL-E 3 outlined the steps of LASIK surgery in text, but the image it created from that text failed to represent LASIK at all. It further degenerated when asked to condense the image, producing unintelligible steps and fictitious ocular anatomy (Figure 4A).

When asked to change the color of anatomy with targeted prompts, both programs struggled. Only 50% (3/6) of the images correctly followed the instruction to “make each iris purple.” This suggests that the programs cannot accurately identify specific anatomy from an image. We further tested this by asking ChatGPT-4o to use our grading system and create its own grading system to evaluate images. It rated Figure 6B a 4.78 out of 5, stating, “It accurately represents the anatomical details, follows the correct procedural steps, and is consistent with medical literature” [13]. This indicates a text-to-picture recognition issue with current AI platforms. Adding a logic-checking mechanism to examine creations for accuracy could significantly improve image creation.

AI's perception of its images is often skewed, resulting in a significant discrepancy between its assessment and that of human examiners. This discrepancy is concerning, as reverse analysis shows that while AI can effectively interpret and describe existing images (image to text), it struggles to generate accurate images from text descriptions (text to image). For example, Figure 5A was given to ChatGPT-4o to describe. The platform recognized it was an image displaying the steps of PRK and listed the steps in the image in detail saying, “This image provides a detailed and labeled cross-sectional view of the eye, highlighting the key anatomical structures and the precise steps involved in the PRK procedure.” Despite understanding and describing other images accurately, AI systems like DALL-E 3 and MiM fail to create visually accurate representations from detailed textual prompts. This highlights a notable gap in AI's capability to translate textual information into precise visual content.

Despite utilizing a subscription-based AI service, we encountered several shortcomings. The prompts were created by clinicians rather than professional prompt engineers, which may have impacted the results. Additionally, it is challenging to manage how the platforms’ algorithms use and process their images. Our sample size of four reviewers limits the power of this study, and we only tested AI's ability to regenerate three refractive procedures. To gain a deeper understanding of AI's capabilities, additional procedures should be tested. Another limitation is reproducibility. Using the same prompts often yields different results, and continued prompts on a singular image can degrade the quality (degeneracy) of the image [11]. We also did not assess or exhaust alternative AI image generation models like Midjourney or Stable Diffusion, to evaluate their capabilities.

## Conclusions

This study demonstrates the lack of accuracy in AI-generated images as it specifically relates to the corneal refractive procedures LASIK, PRK, and SMILE. While the OpenAI platform can create an image that would be recognized by the public as an eye, there is no current educational value in the depictions. The AI’s strengths are in the program's ability to generate creative imagery in an extremely efficient manner. Most of the images, which were vibrant and colorful, took only a few seconds to generate. While AI performs exceptionally well as a language and text-based tool, accurate image generation requires further development.

We believe that AI should be utilized as a tool to increase productivity, much like the advent of the internet and the myriad of opportunities it has enabled. While AI imagery is currently quite inaccurate, we anticipate that with time and development, it will become more frequently used. As AI advances in creating realistic videos and photos, it is essential to remain skeptical about the accuracy of these illustrations. We hope that ongoing research into AI image creation will enhance its accuracy and efficiency, ensuring it becomes a reliable and valuable tool.
